# Risk Factors for Re-Tear of the Meniscus Following Meniscus Repair with Concomitant ACL Reconstruction

**DOI:** 10.3390/jcm14165881

**Published:** 2025-08-20

**Authors:** Kyle R. Gronbeck, Stephen Nystrom, Bryan Perkins, Marc A. Tompkins

**Affiliations:** 1 Department of Emergency Medicine, Sanford Health, Fargo, ND 58104, USA; 2 Aurora Medical Center Oshkosh, Oshkosh, WI 64904, USA; 3 St. Cloud Orthopedics, St. Cloud, MN 56301, USA; 4 TRIA Orthopaedic Center, 8100 Northland Drive, Bloomington, MN 55431, USA; 5 Department of Orthopedic Surgery, University of Minnesota, 2450 Riverside Avenue South, Suite R200, Minneapolis, MN 55455, USA; 6 Gillette Specialty Healthcare, 200 University Av. E, St. Paul, MN 55101, USA

**Keywords:** meniscus repair, meniscal repair, anterior cruciate ligament, ACL, risk factors, pediatric

## Abstract

**Objectives:** To examine the rate of meniscal re-tear in patients with concomitant ACL reconstruction, with specific focus on surgical factors and patient demographic factors. **Methods:** A retrospective chart review was performed on all patients who underwent meniscal repair with concomitant ACL reconstruction at our institution over a seven-year period. Demographic and case variables were assessed, including sex, age, height, weight, BMI, medial versus lateral repairs, ACL graft type, ACL reconstruction technique, meniscus repair technique, and post-operative weight bearing status. Failure of repair was defined as need for repeat surgery on the same meniscus. **Results:** There were 191 patients included in the study; of those 118 did not need further surgery on the meniscus at a minimum of 2 years post operation while 73 did have a re-operation on the same meniscus (rate of failure 38.2%). There were significant differences between re-operation and non-re-operation groups based on ACL graft type (54% failure for allograft vs. 30/23% failure for both autograft cohorts) and meniscal repair side (46% re-tear rate for medial meniscus vs. 17% for lateral meniscus). The pediatric (under 18 years old) cohort included 57 patients; 28 patients required additional meniscal surgery and 29 did not (rate of re-operation 49%). **Conclusions:** The overall failure rate of meniscus repair was nearly 40%. Risk factors for re-tear of the meniscus were repair of the medial meniscus and allograft usage for ACL reconstruction. The rate of re-tear in patients under 18 was nearly 50%, which is higher than in the adult population.

## 1. Introduction

Approximately 1 million meniscus surgeries are performed annually and there are approximately 200,000 anterior cruciate ligament (ACL) tears a year in the United States [1,2,3]. Meniscus tears commonly occur with ACL tears; rates of concomitant meniscal and ACL injuries range from below 50% to over 80% and become even more likely with delayed surgery following ACL injury and in older patients [2,4,5]. Concomitant meniscus repair with ACL reconstruction has seen an increase in occurrence, with meniscus surgeries being performed in up to 50% of ACL surgeries and specifically meniscus repair in up to 35% [6].

Repairing the meniscus is known to produce better meniscal function than a partial meniscectomy, and success of meniscus repair is thought to be high with concomitant ACL reconstruction [7,8,9,10,11]. Some studies have reported success rates, defined as not requiring reoperation up to 6 years post operation, as high as >90% in stable meniscal tears or in menisci repaired concomitantly with ACLs [12,13,14,15]. The long-term results of a meniscus repair may be worse in an isolated meniscus tear compared to when it is performed with an ACL reconstruction [2,16]. Meniscal repairs performed in conjunction with ACL reconstruction carry a 7% absolute and 42% relative risk reduction of re-operation after 2 years compared with isolated meniscal repair.

Most studies about meniscus repair with concomitant ACL reconstruction have looked at outcomes and rates of re-tear/failure [8,15,17]. There is less research on factors involved in failure of meniscus repair in the setting of concomitant ACL reconstruction. This study aimed to look at the rate of meniscal re-tear in patients with concomitant ACL reconstruction, with specific focus on surgical factors of medial versus lateral repairs, ACL graft type, ACL reconstruction technique, meniscus repair technique, and post-operative weight bearing status, as well as demographic factors of sex, age, height, weight, or BMI. A secondary purpose of the study was to compare the pediatric and adult populations; equal to or less than 18 years of age and over 18 years of age. The hypothesis was that none of the surgical or demographic factors would be associated with higher rates of re-operation, and there would be no difference in risk of re-operation between pediatric patients and adults.

## 2. Material and Methods

Our Institutional Review Board reviewed and approved this study under federal guidelines 45 CFR Part 46.110 category (5), research involving no more than minimal risk. A retrospective chart review was performed on all patients who underwent meniscal repair surgery at our institution over an 8 year period, with minimum of two years since the index surgery. Patients were identified using CPT^®^ codes 29882 and 29883 for meniscal repair. All patients who underwent meniscal repair at the same time as concomitant ACL reconstruction were included. Only patients from surgeons who performed at least 5 meniscus repairs with concomitant ACL reconstruction per year were considered. Patients with any other concomitant procedures performed, except contralateral partial meniscectomy or chondroplasty, were excluded.

Demographic variables recorded were height, weight, BMI, age at surgery, and sex. Case variables recorded were: classification of tear type (vertical, radial, horizontal, root); acuity of the tear (acute or chronic as defined by the operating surgeon); repair side (medial or lateral meniscus), repair location within the meniscus (posterior horn, body, or anterior horn); repair technique (inside out, all inside); ACL graft type (patellar tendon autograft, hamstring autograft, allograft), ACL reconstruction technique (two-incision, transtibial, anteromedial), concomitant procedures (including ligament reconstruction, chondroplasty, or partial meniscectomy on the other meniscus in the same knee that was not repaired), post-operative weight bearing status, and meniscal re-operations. Each chart review, including review of the operative report and operative images, was completed by two observers. Questions of tear type, tear location, or otherwise were resolved by consensus.

All patients for whom there was no recorded evidence of re-operation in the chart review were contacted by mail or telephone. The patients were initially contacted by mail. If there was no response, then this was followed by 3 phone calls over the course of 3 weeks or until a response was obtained. At the time of contact, the patients were asked whether they had had surgery again on the same meniscus.

All surgeons used a similar rehabilitation protocol; the protocol delineates expected recovery progression in range of motion, strength, and types of exercises over time from surgery. Regardless of weight bearing status, patients were placed into a brace and restricted in range of motion from 0 to 90 degrees for the first 6 weeks postoperatively. Postoperative weight bearing status was at the discretion of the surgeon. Patients who were allowed full weight bearing were instructed to lock their brace in full extension during weight bearing activities for the first 6 weeks. The protocol was provided to the physical therapist. The rehabilitation was expected to take 4–6 months. The number of in person physical therapy sessions was determined by the therapist.

Failure of repair was defined as the need for repeat surgery on the same meniscus. Independent sample *t*-tests were performed between the patients who did and did not have re-operation for age at surgery, height, weight, and BMI. Chi-square tests were performed to determine if there was an effect on failure of the meniscal repair based on sex, repair side, repair technique, ACL graft type, ACL reconstruction technique, and post-operative weight bearing status. Results were considered significant at *p* ≤ 0.05. The same comparisons were also performed for pediatric (<18 years) and adult (18+) groups.

## 3. Results

### 3.1. Full Cohort

The selection criteria yielded a total of 398 consecutive patients undergoing meniscal repair and concomitant ACL reconstruction that were eligible for inclusion in the study; the cases were from 8 sports medicine fellowship trained surgeons. There were 191 patients who either had evidence of further surgery on the same meniscus in the chart or who responded after being contacted by study authors; these 191 were therefore included in the study. Of these patients, 118 did not need further surgery on the meniscus up to 2 years post operation while 73 did have a re-operation on the same meniscus (rate of failure 38.2%). Two hundred and seven patients were contacted at all of the time points, but did not respond. Of the patients who were contacted but did not respond, only two of them were in the pediatric group (under the age of 18).

All tears in the 191 patients were vertical, peripheral tears of the posterior horn and/or meniscal body. All but 10 of the tears were considered to be acute. There were no significant differences between groups (re-operation and no re-operation) based on demographics: age, height, weight, BMI, and sex (Table 1 and Table 2).

There were significant differences between groups for ACL graft type (*p* = 0.01) and meniscal repair side (*p* < 0.001). Patients with allograft were noted to have a higher rate of re-operation and patients with medial meniscal repairs were noted to have a higher rate of re-operation. There were no significant differences for meniscal repair technique, ACL reconstruction technique, or post-operative weight bearing status (Table 3).

ACL graft type was assessed in each of the meniscus location categories (medial vs. lateral). For medial meniscus repairs, the re-tear rate was 63% when an allograft was used for the ACL reconstruction. This was statistically significant when compared to the hamstring and patellar tendon autograft (*p* = 0.02). This was not the case for the lateral meniscus. Rate of re-tear for the allograft in the lateral meniscus was 18%. There was no statistical significance when compared to patellar tendon and hamstring autograft (*p* = 0.99).

### 3.2. 18+ Age Cohort

This cohort included 134 patients with 89 not requiring additional surgery and 45 requiring additional surgery (rate of re-operation 34%). In this group, surgery on the medial meniscus (*p* = 0.003) and the use of allograft (*p* = 0.01) were also significantly associated with higher risk of re-operation. The other surgical factors were not associated with higher risk of re-operation (Table 4).

### 3.3. Age 18 and Under Cohort

This cohort included 57 patients; 28 patients who required additional meniscal surgery and 29 that did not (rate of re-operation 49%). In the under 18 group, none of the surgical factors were associated with higher risk of re-operation (Table 5).

## 4. Discussion

The most important findings from this study were that the risk of meniscus re-operation in concomitant ACL reconstruction patients is noteworthy; this study found higher re-operation rates than many previous reports with an overall failure rate for all included patients approaching 40%. Surgical parameters associated with higher risk for re-operation were found to be medial meniscus repairs and allograft usage for ACL reconstruction. The rate of re-operation for the pediatric population in this cohort was higher than in adults at nearly 50%.

Studies have shown that failure rates in meniscus repairs with concomitant ACL reconstructions are low, including as low as 10% [10,15,18]. Meniscus repairs, in variable settings (not just with concomitant ACL reconstruction), have been shown to have a wide range of failure rates from acuity% to upwards of 90% [19,20,21]. A meta-analysis, with pooled data, demonstrated a 20–24% failure rate in meniscus repairs (all techniques, ACL deficient, and ACL intact knees) [12]. A study by Matsushita et al. where they performed second look arthroscopy after concomitant ACL reconstruction and meniscus repair, demonstrated that 22% of patients had a meniscal re-tear and another 22% had incomplete healing; they did not describe how many of these patients had a re-operation on the re-torn or partially healed meniscus [22]. The rate of re-operation in our cohort is higher than most of these studies, with a failure rate of 38.2%. The rate of re-operation in our cohort was even higher in patients under the age of 18 (49%). Primarily adult patients were included in many previous studies, so the higher rate in the pediatric population within our cohort may skew the results higher in our study. While we were only able to capture data on a little less than 50% of the eligible patients, it is still notable that there were so many re-operations. Even if there had been no patients lost to follow up and no other patients had undergone a re-operation, the rate of re-operation would still be over 18% (73/398 patients). So, the true rate in our population of 398 patients identified during the study period is 18% or higher. It is important for surgeons to be aware of this and to counsel patients that there is a reasonable chance of re-operation following meniscal repair, even in the setting of concomitant ACL reconstruction.

It is unclear why adolescents would fail at a higher rate with concomitant ACL reconstruction. This may be due to changing biomechanics and neuromuscular function during growth, type of physical activity stress on the knee, and increased levels of athletic activity causing more exposures and therefore opportunities for re-injury. Previous studies on meniscus repair, specifically in the pediatric population, have also demonstrated variable failure rates, and it is likely that the amount of activity has something to do with failure risk [6,23,24]. The rates are likely lower when there is a concomitant ACL reconstruction, but the few studies currently available still suggest that there is likely a higher failure rate in the pediatric population than failure rates reported in the adult population [6,23,24]. Our data adds to the evidence that the failure risk may be even higher in the pediatric population, and this is important information for surgeons counseling pediatric patients and their families.

Based on our data, the rate of re-tear is impacted by which meniscus is involved. Our study found that 46% of medial meniscus repairs fail at a minimum of two years post operation as compared to 17% of lateral menisci. In the meta-analysis by Nepple et al., the rate of failure was similar between the medial and the lateral meniscus [12]. Other studies, including the study by Paxton et al. and a study from the MOON Group, however found a higher failure rate in medial meniscus repairs, consistent with our data [9,25]. Even though the medial meniscus has a larger vascular supply, it is possible the worse rates of re-tear are related to the medial side of the meniscus being anchored more tightly to the tibial plateau and being subjected to higher biomechanical loads [26]. This may be exacerbated in an ACL tear setting when there is impaired knee stability and the medial meniscus will assume more responsibility for stability [27,28].

The rate of meniscal re-operation was impacted by which graft type was used in our study with the rate of meniscal repair failure when using allograft being 20% higher than in hamstring and patellar tendon autograft. The study from the MOON group also found use of allograft had a 2.3 times higher rate of meniscus reoperation than if BTB autograft was used for the ACL [25]. Multiple studies on ACL reconstruction outcomes based on graft type have shown allografts have a higher rate of ACL reconstruction failure than autografts [29,30,31,32]. Our study did not evaluate the rates of ACL reconstruction failure in the cohort, but it is possible the allograft is not providing adequate ACL function in as many knees as autograft options (whether requiring ACL re-operation or not), leading to greater risk of re-injury to the meniscus in addition to the ACL. The study by Matsushita et al. found that patients with a meniscus re-tear were more likely to have a positive pivot shift test supporting the idea that inadequate ACL function increases the risk of meniscal re-tear [22].

The rate of re-operation in our cohort was not impacted by the technique used for ACL reconstruction (anteromedial, transtibial, or two incision). While biomechanical studies have suggested some benefit from independent femoral tunnel drilling techniques, clinical studies have failed to elicit a difference [33,34,35]. These findings may carry over to the risk of meniscal re-tear. Additionally, meniscus repair failure was not affected by the meniscal repair technique (all-inside with FasT-Fix ^TM^ device (Smith & Nephew, Andover, MA, USA), or inside-out); the rate of re-operation was 38% for all-inside and 37% for inside out. The fact that there was no difference between the two techniques is similar to what is reported in other studies looking at only isolated meniscus repairs [36,37].

There are many limitations of this study. The first is that it is a retrospective study, which resulted in a significant portion of the potential patients not being included because we were not able to reach them greater than two years post-operatively, despite repeated attempts. As noted previously, this likely skews the rate of re-operation higher. Despite this, it is still a large enough number of patients included that we felt it was felt important to report the data that there is certainly still a notably high risk of re-operation, particularly in the pediatric population where we had minimal loss to follow up. The retrospective nature also does not make it possible to control for how rehabilitation was conducted. Incorporating data collected from 8 separate surgeons at the same institution also does not make it possible to standardize skill and overall surgical technique. In addition, surgeon description of acute vs. chronic tear is somewhat subjective. Despite the inclusion of patients from multiple surgeons, however, we do feel this is representative of a broader population of patients and therefore may be more realistic for the general population. Despite the numbers of included patients, it is possible that the study is underpowered to detect a difference in some of the analyses. A final limitation is that we do not know the number of ACL re-tears which could also impact the number of meniscal re-operations.

## 5. Perspective

The risk of meniscal reoperation when meniscal repair was performed with concomitant ACL reconstruction was nearly 40%. The risk factors for re-tear of the meniscus were repair of the medial versus the lateral meniscus and allograft usage for the ACL reconstruction. The rate of re-tear in patients under 18 was nearly 50%, which is higher than in the adult population.

## Figures and Tables

**Table 1:** Demographic information for patients with or without an additional meniscus surgery.

Demographic	Additional Meniscus Surgery	*n*	Mean	Standard Deviation	*p*-Value
Age at surgery (years)	Yes	73	23.4	10.0	*p* = 0.051
	No	118	26.3	9.91	
Height (m)	Yes	67	1.73	0.11	*p* = 0.37
	No	111	1.71	0.12	
Weight (kg)	Yes	70	77.0	17.2	*p* = 0.96
	No	113	76.9	17.6	
BMI (kg/m^2^)	Yes	67	25.3	4.44	*p* = 0.61
	No	112	25.7	4.63	

Abbreviations: BMI, Body Mass Index.

**Table 2:** Sex for patients with or without an additional meniscus surgery.

		Additional Meniscus Surgery		Total	*p*-Value
		No	Yes		
Sex	Female	59	39	98	*p* = 0.66
	Male	59	34	93	
Total		118	73	191	

**Table 3:** Raw data, rates of re-operation, and statistical significance (*p*-values) for the 4 different surgical categories: ACL technique, meniscus tear location, meniscus repair technique, and ACL graft type.

Surgical Data Category	Sub-Category	Re-Operation		Total (*n*)	Rate of Re-Operation	*p*-Value
		No (*n*)	Yes (*n*)			
ACL technique	Trans-tibial	41	27	68	40%	*p* = 0.75
	Two incision	27	19	46	41%	
	Antero-medial	50	27	77	35%	
Meniscus location	Medial meniscus	75	64	139	46%	*p* < 0.001
	Lateral meniscus	43	9	52	17%	
Meniscus repair technique	Inside out	32	20	52	38%	*p* = 0.97
	All inside	86	53	139	37%	
ACL graft type	Hamstring autograft	43	20	63	32%	*p* = 0.01
	BTB autograft	48	21	69	30%	
	Allograft	27	32	59	54%	
Weight bearing status	Non-weight bearing	25	19	44	43%	*p* = 0.44
	Weight bearing as tolerated	93	54	147	37%	
Total applied to each surgical data category		118	73			

Abbreviations: ACL, Anterior Cruciate Ligament.

**Table 4:** Data for the 18+ group, rates of re-operation, and statistical significance (*p*-values) for the 4 different surgical categories: ACL technique, meniscus tear location, meniscus repair technique, and ACL graft type.

Surgical Data Category	Sub-Category	Re-Operation		Total (*n*)	Rate of Re-Operation	*p*-Value
		No (*n*)	Yes (*n*)			
ACL technique	Trans-tibial	33	18	51	35%	*p* = 0.33
	Two incision	14	11	25	44%	
	Antero-medial	42	16	58	28%	
Meniscus location	Medial meniscus	54	39	93	42%	*p* < 0.001
	Lateral meniscus	35	6	41	15%	
Meniscus repair technique	Inside out	22	11	33	33%	*p* = 0.97
	All inside	67	34	101	34%	
ACL graft type	Hamstring autograft	31	9	40	23%	*p* = 0.01
	BTB autograft	34	12	46	26%	
	Allograft	24	24	48	50%	
Totals applied to each surgical data category (*n*)		89	45			

Abbreviations: ACL, Anterior Cruciate Ligament.

**Table 5:** Data for the under 18 group, rates of re-operation, and statistical significance (*p*-values) for the 4 different surgical categories: ACL technique, meniscus tear location, meniscus repair technique, and ACL graft type.

Surgical Data Category	Sub-Category	Re-Operation		Total (*n*)	Rate of Re-Operation	*p*-Value
		No (*n*)	Yes (*n*)			
ACL technique	Trans-tibial	8	9	17	32%	*p* = 0.43
	Two incision	13	8	21	29%	
	Antero-medial	8	11	19	39%	
Meniscus location	Medial meniscus	21	25	46	54%	*p* = 0.18
	Lateral meniscus	8	3	11	28%	
Meniscus repair technique	Inside out	10	9	19	47%	*p* = 0.85
	All-inside	19	19	38	50%	
ACL graft type	Hamstring autograft	12	11	23	48%	*p* = 0.18
	BTB autograft	14	9	23	39%	
	Allograft	3	8	11	73%	
Totals applied to each surgical data category (*n*)		29	28			

Abbreviations: ACL, Anterior Cruciate Ligament.

## Data Availability

The original contributions presented in this study are included in the article. Further inquiries can be directed to the corresponding author(s).

## References

[1] Beck N.A., Lawrence J.T.R., Nordin J.D., DeFor T.A., Tompkins M. (2017). ACL tears in school-aged children and adolescents over 20 years. Pediatrics.

[2] DeFroda S.F., Yang D.S., Donnelly J.C., Bokshan S.L., Owens B.D., Daniels A.H. (2020). Trends in the surgical treatment of meniscal tears in patients with and without concurrent anterior cruciate ligament tears. Phys. Sport..

[3] Jones J.C., Burks R., Owens B.D., Sturdivant R.X., Svoboda S.J., Cameron K.L. (2012). Incidence and risk factors associated with meniscal injuries among active-duty US military service members. J. Athl. Train..

[4] Erard J., Cance N., Shatrov J., Fournier G., Gunst S., Ciolli G., Servien E. (2023). Delaying ACL reconstruction is associated with increased rates of medial meniscal tear. Knee Surg. Sports Traumatol. Arthrosc..

[5] Kilcoyne K.G., Dickens J.F., Haniuk E., Cameron K.L., Owens B.D. (2012). Epidemiology of meniscal injury associated with ACL tears in young athletes. Orthopedics.

[6] Block A.M., Eisenberg M.T., Inclan P.M., Nepple J.J. (2022). Treatment Trends in Meniscal Pathology in the Setting of Concomitant ACL Injuries in Pediatric and Young Adult Patients: An Insurance Database Study. Am. J. Sports Med..

[7] Lutz C., Dalmay F., Ehkirch F.P., Cucurulo T., Laporte C., Le Henaff G., Potel J.F., Pujol N., Rochcongar G., Salledechou E. (2015). Meniscectomy versus meniscal repair: 10 years radiological and clinical results in vertical lesions in stable knee. Orthop. Traumatol. Surg. Res..

[8] Melton J.T., Murray J.R., Karim A., Pandit H., Wandless F., Thomas N.P. (2011). Meniscal repair in anterior cruciate ligament reconstruction: A long-term outcome study. Knee Surg. Sports Traumatol. Arthrosc..

[9] Paxton E.S., Stock M.V., Brophy R.H. (2011). Meniscal repair versus partial meniscectomy: A systematic review comparing reoperation rates and clinical outcomes. Arthroscopy.

[10] Wright R.W., Huston L.J., Haas A.K. (2023). Ten-Year Outcomes of Second-Generation, All-Inside Meniscal Repair in the Setting of ACL Reconstruction. J. Bone Jt. Surg. Am..

[11] Xu C., Zhao J. (2015). A meta-analysis comparing meniscal repair with meniscectomy in the treatment of meniscal tears: The more meniscus, the better outcome?. Knee Surg. Sports Traumatol. Arthrosc..

[12] Nepple J.J., Block A.M., Eisenberg M.T., Palumbo N.E., Wright R.W. (2022). Meniscal Repair Outcomes at Greater Than 5 Years: A Systematic Review and Meta-Analysis. J. Bone Jt. Surg. Am..

[13] Petersen W., Karpinski K., Bierke S., Müller Rath R., Häner M. (2022). A systematic review about long-term results after meniscus repair. Arch. Orthop. Trauma. Surg..

[14] Schweizer C., Hanreich C., Tscholl P.M., Blatter S., Windhager R., Waldstein W. (2024). Meniscal Repair Outcome in 3829 Patients With a Minimum Follow-up From 2 Years Up to 5 Years: A Meta-analysis on the Overall Failure Rate and Factors Influencing Failure. Am. J. Sports Med..

[15] Westermann R.W., Wright R.W., Spindler K.P., Huston L.J., Wolf B.R. (2014). Meniscal repair with concurrent anterior cruciate ligament reconstruction: Operative success and patient outcomes at 6-year follow-up. Am. J. Sports Med..

[16] Pelletier S., Djebara A., Freychet B., Carnessechi O., Graveleau N., Louis M.-L., Benad K., Praz C., Maroteau G., Badr S. (2023). Medial meniscal repair in stable knees: Survival rate and risk factors for failure at a minimum of 5 years. Orthop. Traumatol. Surg. Res..

[17] Jackson T., Fabricant P.D., Beck N., Storey E., Patel N.M., Ganley T.J. (2019). Epidemiology, Injury Patterns, and Treatment of Meniscal Tears in Pediatric Patients: A 16-Year Experience of a Single Center. Orthop. J. Sports Med..

[18] Lee G.R., Diduch D.R. (2005). Deteriorating outcomes after meniscal repair using the meniscus arrow in knees undergoing concurrent anterior cruciate ligament reconstruction. Increased failure rate with long-term follow-up. Am. J. Sports Med..

[19] Alhamdi H., Foissey C., Vieira T.D., Sonnery-Cottet B., Rajput V., Bahroun S., Thaunat M. (2023). High failure rate after medial meniscus bucket handle tears repair in the stable knee. Orthop. Traumatol. Surg. Res..

[20] Tripon M., Praz C., Ferreira A., Drigny J., Reboursière E., Hulet C. (2023). Clinical outcome of iterative meniscal suture after ACL reconstruction at a minimum of 2 years’ follow-up. Orthop. Traumatol. Surg. Res..

[21] Tuckman D.V., Bravman J.T., Lee S.S., Rosen J.E., Sherman O.H. (2006). Outcomes of meniscal repair: Minimum of 2-year follow-up. Bull. Hosp. Jt. Dis..

[22] Matsushita T., Nagai K., Araki D., Tanaka T., Matsumoto T., Nishida K., Kuroda R. (2017). Factors associated with the status of meniscal tears following meniscal repair concomitant with anterior cruciate ligament reconstruction. Connect. Tissue Res..

[23] Accadbled F., Cassard X., Sales de Gauzy J., Cahuzac J.P. (2007). Meniscal tears in children and adolescents: Results of operative treatment. J. Pediatr. Orthop. B.

[24] Shieh A.K., Edmonds E.W., Pennock A.T. (2016). Revision meniscal surgery in children and adolescents: Risk factors and mechanisms for failure and subsequent management. Am. J. Sports Med..

[25] Salem H.S., Huston L.J., Zajichek A., McCarty E.C., Vidal A.F., Bravman J.T., Wright R.W. (2021). Anterior Cruciate Ligament Reconstruction With Concomitant Meniscal Repair: Is Graft Choice Predictive of Meniscal Repair Success?. Orthop. J. Sports Med..

[26] Arnoczky S.P., Warren R.F. (1982). Microvasculature of the human meniscus. Am. J. Sports Med..

[27] Peltier A., Lording T., Maubisson L., Ballis R., Neyret P., Lustig S. (2015). The role of the meniscotibial ligament in posteromedial rotational knee stability. Knee Surg. Sports Traumatol. Arthrosc..

[28] Walker P.S., Arno S., Bell C., Salvadore G., Borukhov I., Oh C. (2015). Function of the medial meniscus in force transmission and stability. J. Biomech..

[29] Cvetanovich G.L., Mascarenhas R., Saccomanno M.F., Verma N.N., Cole B.J., Bush-Joseph C.A., Bach B.R. (2014). Hamstring autograft versus soft-tissue allograft in anterior cruciate ligament reconstruction: A systematic review and meta-analysis of randomized controlled trials. Arthroscopy.

[30] Kaeding C.C., Aros B., Pedroza A., Pifel E., Amendola A., Andrish J.T., Spindler K.P. (2011). Allograft versus autograft anterior cruciate ligament reconstruction: Predictors of failure from a MOON prospective longitudinal cohort. Sports Health.

[31] Lenehan E.A., Payne W.B., Askam B.M., Grana W.A., Farrow L.D. (2015). Long-term outcomes of allograft reconstruction of the anterior cruciate ligament. Am. J. Orthop..

[32] Mascarenhas R., Erickson B.J., Sayegh E.T., Verma N.N., Cole B.J., Bush-Joseph C., Bach B.R. (2015). Is there a higher failure rate of allografts compared with autografts in anterior cruciate ligament reconstruction: A systematic review of overlapping meta-analyses. Arthroscopy.

[33] Chalmers P.N., Mall N.A., Cole B.J., Verma N.N., Bush-Joseph C.A., Bach B.R. (2013). Anteromedial versus transtibial tunnel drilling in anterior cruciate ligament reconstructions: A systematic review. Arthroscopy.

[34] Liu A., Sun M., Ma C., Chen Y., Xue X., Guo P., Yan S. (2017). Clinical outcomes of transtibial versus anteromedial drilling techniques to prepare the femoral tunnel during anterior cruciate ligament reconstruction. Knee Surg. Sports Traumatol. Arthrosc..

[35] Zhang Y., Xu C., Dong S., Shen P., Su W., Zhao J. (2016). Systemic review of anatomic single- versus double-bundle anterior cruciate ligament reconstruction: Does femoral tunnel drilling technique matter?. Arthroscopy.

[36] Fillingham Y.A., Riboh J.C., Erickson B.J., Bach B.R., Yanke A.B. (2017). Inside-Out Versus All-Inside Repair of Isolated Meniscal Tears: An Updated Systematic Review. Am. J. Sports Med..

[37] Grant J.A., Wilde J., Miller B.S., Bedi A. (2012). Comparison of inside-out and all-inside techniques for the repair of isolated meniscal tears: A systematic review. Am. J. Sports Med..

